# Using methods to extend inferences to specific target populations to improve the precision of subgroup analyses

**DOI:** 10.1016/j.jclinepi.2025.111716

**Published:** 2025-02-07

**Authors:** Michael Webster-Clark, Anthony A. Matthews, Alan R. Ellis, Alan C. Kinlaw, Robert W. Platt

**Affiliations:** a Division of Public Health Sciences, Wake Forest University School of Medicine, Winston-Salem, NC; b Department of Clinical Epidemiology, McGill University, Montreal, Quebec, Canada; c Department of Epidemiology, University of North Carolina at Chapel Hill, Chapel Hill, NC, USA; d Unit of Epidemiology, Institute of Environmental Medicine, Karolinska Institutet, Stockholm, Sweden; e School of Social Work, North Carolina State University, Raleigh, NC, USA

**Keywords:** External validity, Subgroup, Colorectal cancer, Transportability, Generalizability, Standardization, Randomized controlled trials

## Abstract

**Objectives:**

While subgroup analyses are common in epidemiologic research, restriction to subgroup members can yield imprecise estimates. We aimed to demonstrate how methods extending inferences to external targets improve precision of subgroup estimates under the major assumption effects differ between subgroup members and nonmembers due to measured effect measure modifiers (EMMs) and membership is independent of the effect after conditioning on EMMs.

**Study Design and Setting:**

We applied this approach in the Panitumumab Randomized Trial in Combination with Chemotherapy for Metastatic Colorectal Cancer to Determine Efficacy. Assuming Hispanic vs non-Hispanic ethnicity was independent of the effect conditional on measured EMMs, we weighted non-Hispanic White participants to resemble Hispanic participants in EMMs, assigned Hispanic participants weights of 1, and estimated weighted 9-month progression-free survival differences (PFSDs) with 95% confidence limits from 2000 bootstraps. We also explored outcome-based approaches. Finally, we examined a situation where the method generates biased estimates (targeting participants with mutant-type Kirsten rat sarcoma virus (KRAS), which determines efficacy).

**Results:**

While the Hispanic participant-only analysis estimated a 9-month panitumumab PFSD of −7.1% (95% CI 32%, 19%), the weighted combined estimate targeting Hispanic participants was much more precise (−3.7%, 95% CI: −16%, 9.2%). Other analytic approaches yielded similar results. Meanwhile, the weighted combined estimate targeting mutant-type KRAS participants appeared biased (−2.2%, 95% CI: −7.5%, 3.3%) vs the subgroup-only estimate (−11%, 95% CI: −18%, −2.3%).

**Conclusion:**

While extending inferences from study populations to specific targets can improve the precision of estimates in small subgroups, violating key assumptions creates bias for many subgroups of interest.

## Plain Language Summary

Understanding the benefits and harms in specific subgroups of patients is an important part of epidemiologic and public health research. Unfortunately, commonly used methods to do subgroup analyses can result in estimates with lots of uncertainty. Repurposing methods that have traditionally been used to “generalize” or “transport” effect estimates from specific studies to the types of patients more likely to be encountered in the real world could be used to obtain more informative estimates in subgroups without ignoring differences between different types of patients. In this project, we applied this strategy to the Panitumumab Randomized Trial in Combination with Chemotherapy for Metastatic Colorectal Cancer to Determine Efficacy (PRIME) to create much less variable estimates of the treatment effect in Hispanic participants without ignoring the fact that there were more Hispanic participants with a tumor variation that changed the effect of treatment. On the other hand, when we tried to apply this strategy to improve estimates in patients with that tumor variation, we ended up with a misleading effect estimate. While these methods can reduce uncertainty about the benefits of treatment in specific subgroups interesting to researchers, they can result in incorrect subgroup estimates when their assumptions are violated.

## 1. Introduction

Subgroup analyses in clinical trials and epidemiologic research are essential to identify populations to prioritize for interventions and assess policy impacts on key groups. Typically, investigators separate populations based on characteristics, estimate separate causal effects (hereafter, “effects”), then compare estimates [1]. If estimates differ statistically or clinically, investigators declare the subgroup-defining characteristics important effect measure modifiers (EMMs) (a version of “effect modifiers” or “effect moderation,” specific to a scale like the risk difference or risk ratio) [2–6] and rely on subgroup estimates. If estimates are similar, full-population estimates are treated as the best estimate of subgroup effects. Small sample sizes and random chance can cause false positives and negatives [7,8], leading to the underuse and overuse of interventions and policies. Even after identifying EMMs, subgroup estimates can be imprecise and unhelpful for clinical and regulatory decision-making.

Meanwhile, extending inferences from studies to target populations (ie, “transporting” effect estimates) [9–14] has become increasingly common. Inference extending methods estimate effects in target populations using covariate information. Early applications extended inferences from randomized trials like AIDS Clinical Trial Group [15] or RE-LY [16] to real-world US populations. More recently, these methods were applied to multisite trials [17,18], distributed data networks [19], and external subgroups [20]. Another use of these methods is increasing precision of estimates for internal study subgroups of clinical interest when subgroup membership does not modify effects conditional on other measured variables. We demonstrate this approach in the PRIME (National Clinical Trial ID: NCT00364013) [21,22]. We target one subgroup where the method may be valid (Hispanic participants) and one where its assumptions are violated (participants with mutant-type Kirsten rat sarcoma virus [KRAS] tumors).

## 2. Methods

### 2.1. Data

PRIME was a phase III clinical trial (NCT identifier: NCT00364013) randomizing 1183 participants with metastatic colorectal cancer to receive standard of care (12 cycles of oxaliplatin, 5-fluorouracil, and leucovorin, a.k.a. FOLFOX) or FOLFOX with panitumumab [21,22]. After phase II studies suggested benefit in wild-type KRAS participants, analyses were stratified by KRAS variant. In final analyses [21], the panitumumab-containing regimen demonstrated superior median progression-free survival (PFS) only in wild-type KRAS participants. Deidentified data on 935 PRIME trial participants are available on Project Datasphere.

### 2.2. Effect measure modification

#### 2.2.1. Effect measure modifiers (EMMs)

EMMs are variables across which effect measures of interest vary. Populations with differing distributions of EMMs are typically affected differently by identical interventions [3]. While we cannot identify EMMs directly, consistency (eg, the outcomes we observe in the panitumumab group equal the outcomes they would have experienced when assigned to panitumumab, typically met in RCTs absent data tampering) and randomization (or conditional exchangeability, in observational studies) allow estimation of effects from outcomes of study participants and comparing subgroup effects to evaluate EMM. Mathematical definitions of consistency, effect measure modification, and other key terms are in Appendix 1.

A clinical example of an EMM within PRIME is tumor KRAS variant. The benefits of adding panitumumab to FOLFOX varied by KRAS variant on every scale because adding panitumumab benefited some and harmed others. This means 9-month progression-free survival differences (PFSDs) between panitumumab with FOLFOX and FOLFOX-only arms varied between wild-type KRAS and mutant-type KRAS participants. As a result, KRAS variant is an EMM for the PFSD of adding panitumumab to FOLFOX.

#### 2.2.2. Conditional effect measure modifiers (cEMMs)

Unfortunately, identifying EMMs only captures variation within individual variables [23] despite external validity depending on causal relationships between variables [9]. We can incorporate multiple variables into EMM by conditioning before evaluating effect measure modification [24]. A variable is a cEMM when effects vary across levels of a variable even after accounting for other covariates and their interactions with treatment. Identifying cEMM requires comparing effect estimates between subgroup members and nonmembers after causing them to have similar distributions of other covariates through weighting or outcome modeling. Which variables are cEMM for an effect measure can vary for different adjustment sets.

**Figure 1.**
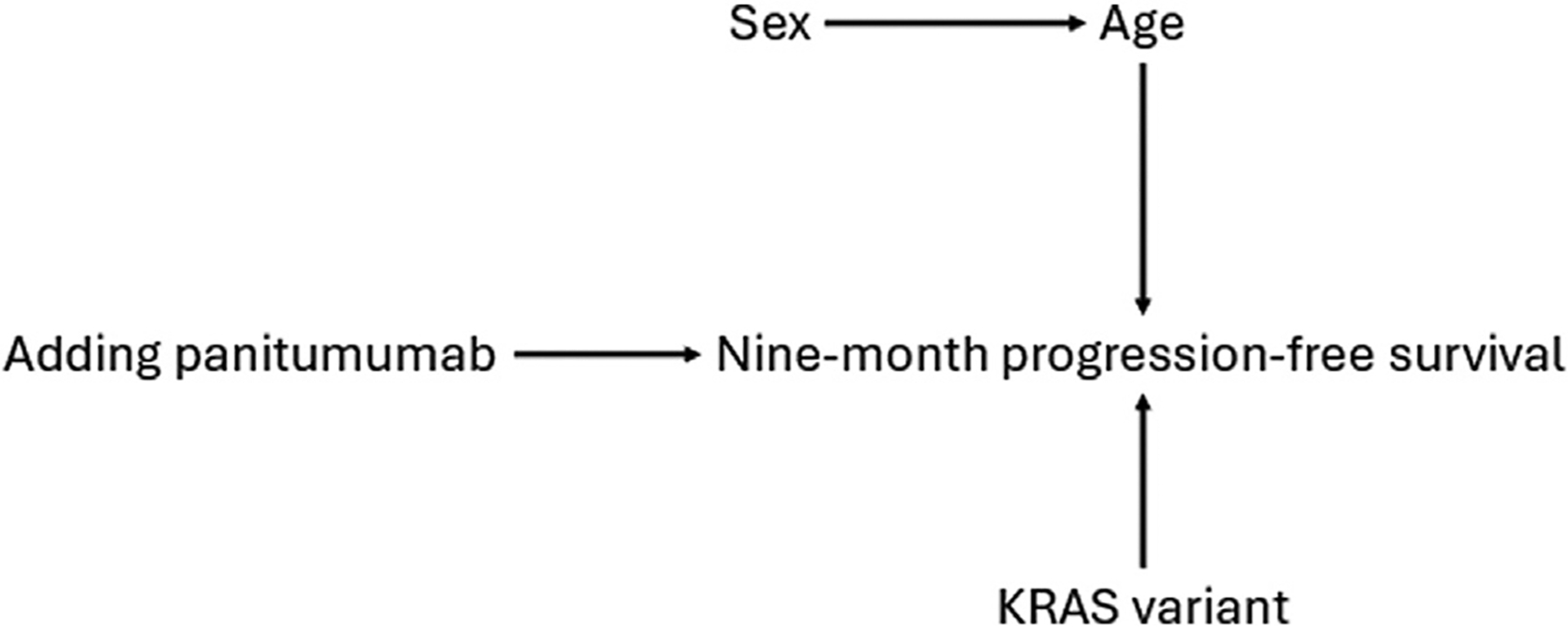
Example directed acyclic graph showing potential associations between the addition of panitumumab to chemotherapy regimens, 9-month progression-free survival, type of KRAS variant, age, and sex in participants with metastatic colon or rectal cancer. Arrows represent causal effects of variables on one another.

Consider a directed acyclic graph of causal relationships between panitumumab, 9-month PFS, age, sex, and KRAS variant (Fig 1). Given age’s open path to the outcome after conditioning on exposure and exposure’s effect on the outcome, age is an EMM on at least one scale [5,6]. Suppose we further know that those over 65 years do not benefit from panitumumab (regardless of KRAS), meaning age modifies effects on every scale. The arrow directly from age into PFS and the lack of arrows from age into KRAS or vice versa means, assuming faithfulness, older age will be associated with PFS conditional on KRAS variant and its modification will remain intact. As a result, age is an EMM (overall) and a cEMM (conditional on KRAS variant).

#### 2.2.3. When EMMs are not cEMMs

Importantly, a variable can be an EMM but not a cEMM under specific conditioning variables [24]. In Figure 1, sex is associated with age (women in the study are older than men). Modification from age “flows” through the association between sex and age, resulting in differing effects in male and female participants [23,25]. This means sex meets our definition of a PFSD EMM and applying full-population estimates to sex subgroups would create bias.

Because the only causal path from sex to PFS goes through age, however, conditioning on age blocks impacts of differing age distributions. This “blocking” means sex is not a cEMM conditional on age and PFSDs would be identical if we accounted for differing age distributions between male and female participants. If the PFSDs were identical, we could “borrow” information from male participants to learn about the PFSD in female participants and vice versa. Fortunately, methods for extending inferences to target populations can account for covariate differences between populations.

### 2.3. Methods for extending inferences and subgroups

#### 2.3.1. Key assumptions

Inference-extending methods have three key assumptions [13,26]: external consistency, meaning the exposure contrast under consideration must not vary between source and target populations; external conditional exchangeability, meaning conditioning on measured covariates must yield equivalent effect estimates in source and target populations (what that means in terms of EMM and cEMM varies by effect measure: eg, the risk difference requires similar distributions of all variables that interact with the treatment on the absolute scale, while the risk ratio requires similar distributions of all variables that interact with the treatment on the ratio scale or are associated with those variables and the outcome) [24]; and external positivity, meaning covariate combinations defining variation in the effect measure for external conditional exchangeability must occur in the source if present in the target.

If these assumptions are met with nonmembers as source and subgroup members as target, inferences from nonmembers can extend to subgroup members. If Figure 1 is true with “female participants” as target and “male participants” as source, we have external consistency because both exposure contrasts are between panitumumab with FOLFOX and FOLFOX-only chemotherapy, external exchangeability because adjusting for a measured variable (age) achieves external validity, and external positivity if there are male and female patients under and over the age of 65 years. We could weight (described below), outcome model, or otherwise account for age differences between male and female participants.

#### 2.3.2. Weighting

**Figure 2.**
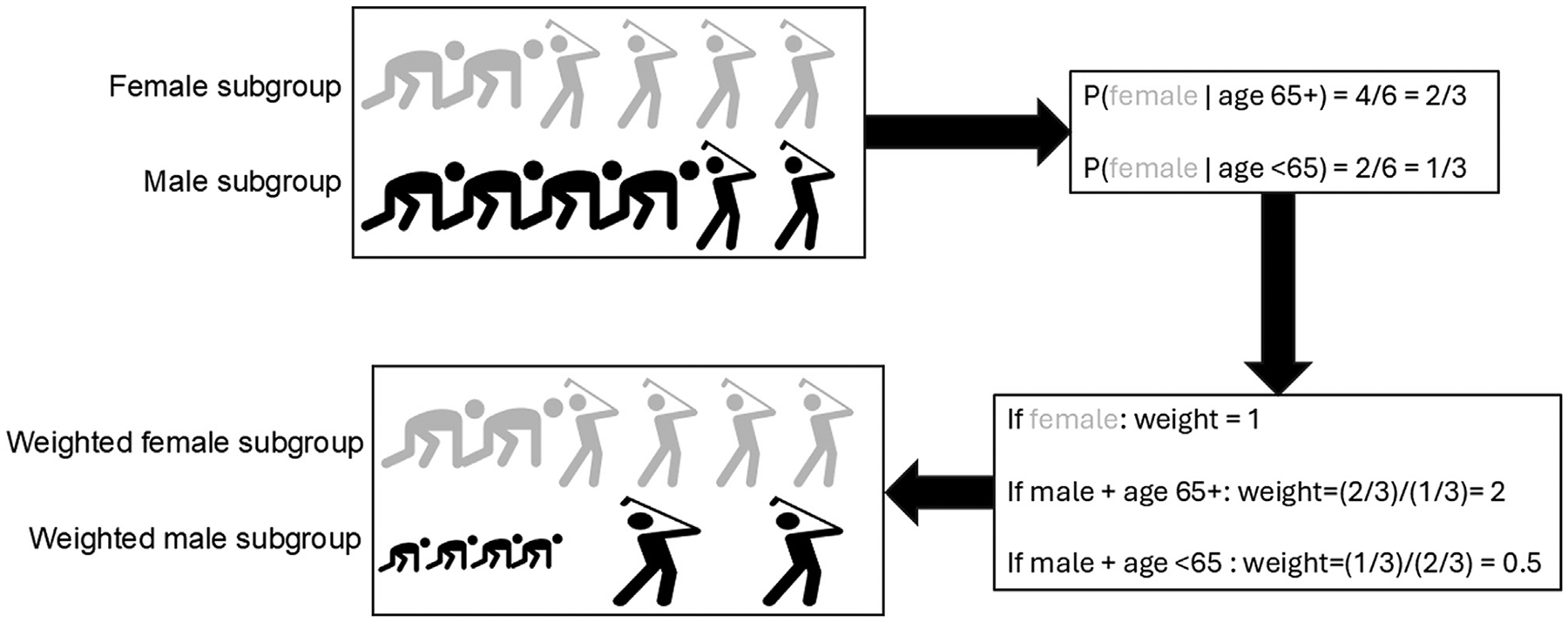
Graphic demonstrating weighting subgroup nonmembers to resemble subgroup members based on suspected effect measure modifiers, using an example reweighting male participants (in black) to resemble female participants (in gray) in terms of age (with younger people represented as running and older people represented as playing golf).

Weighting is one way to balance distributions of multiple variables simultaneously [27–29]. Weighting constructs a “pseudopopulation” with an identical covariate distribution as subgroup members from subgroup nonmembers. Figure 2 illustrates weighting. First, separate (in Fig 2, female participants in gray) and nonmembers (in Fig 2, male participants in black); second, use a predictive model like multivariable logistic regression to estimate the probability of being a subgroup member (in Fig 2, a gray figure) conditional on variables preventing membership from being a cEMM (in Fig 2, age greater than 65 years as represented by golfing); and third, assign nonmembers weights equal to predicted covariate-conditional odds of subgroup membership (odds weights [OW]) and subgroup members weights of 1. This results in populations with similar covariate distributions (in Fig 2, 1/3 of female and weighted male patients are over 65 years). If assumptions are met, analyzing subgroup members and weighted nonmembers estimates the same quantity: the effect in subgroup members. If researchers are concerned about imbalances between arms in baseline characteristics, separate models can be fit to reweight each treatment arm of nonmembers to the full population of subgroup members [16].

#### 2.3.3. Combining estimates

While estimates from subgroup members and weighted nonmembers can and should be reported separately, synthesizing can yield more informative effect estimates [30]. The most straightforward approach to synthesis is analyzing members and weighted nonmembers as one population (hereafter, “combined”). We could also pool estimates using inverse variance weighting (IVW) methods (hereafter, “IVW”) [31]. In simulations (Appendix 2), combined and IVW approaches achieved CI coverage close to the nominal value of 95% with sufficient adjustment sets, though combining was less precise. Omitting necessary EMM from adjustment sets biased weighted nontarget estimates and made performance of the approaches depend on sizes of targets relative to nontargets. When targets were smaller than nontargets, IVW yielded more biased estimates with inferior CI coverage. When targets were larger than nontargets, IVW dominated in bias, precision, and CI coverage.

### 2.4. Applied example

#### 2.4.1. Rationale for selecting the PRIME trial

PRIME measured a well-understood biologically plausible mechanism of effect heterogeneity (KRAS variant) and other characteristics defining subgroups of interest. This setting provides an opportunity to explore when inference-extending methods may be beneficial (when subgroup membership is an EMM but may not be a cEMM, as with Hispanic ethnicity) or generate biased estimates (when subgroup membership is a cEMM, as with mutant-type KRAS).

#### 2.4.2. Targeting Hispanic participants

First, we used non-Hispanic White participants to improve precision of effect estimates in Hispanic participants. Hispanic participants and non-Hispanic White participants differed in KRAS (which varies across race and ethnic groups due to gene expression) [32]. While there is known heterogeneity between Hispanic patients and non-Hispanic White patients in cancer outcomes among treated patients (implying EMM on at least one scale), it is hypothesized to result from poorer health-care access and financial toxicity [33], which are attenuated in trial participants. Possible influence of later diagnosis is also reduced in a randomized trial of patients with metastatic cancer and measured performance status. As a result, there are differences between Hispanic and non-Hispanic White participants in EMMs, but potential conditional effect measure modification of Hispanic ethnicity may be limited.

Project Datasphere includes treatment, covariate, and outcome data for 42 Hispanic participants and 795 non-Hispanic White participants from PRIME. While 40% (17/42) of Hispanic participants had wild-type KRAS, 60% (479/795) of non-Hispanic White participants had wild-type KRAS. This suggests Hispanic participants would benefit less from panitumumab than non-Hispanic White participants. It is worth accounting for additional potential modifiers in case they also threaten external validity, including age over 65 years, colon vs rectal cancer, liver metastases, performance status (a measure of functioning in cancer patients), and sex.

We estimated the probability of being a Hispanic participant with multivariable logistic regression conditional on these variables, fitting separate models to make panitumumab with FOLFOX and FOLFOX-only arms non-Hispanic White participants possess the same covariate distributions as all Hispanic participants. We assigned non-Hispanic White participants OW equal to their covariate-conditional odds of being a Hispanic participant based on measured covariates. We multiplied weights by 0.5 to create a weighted non-Hispanic white population similar in size to the Hispanic participants (without this multiplication, using separate weights for each arm would result in a weighted non-Hispanic White population twice the size of the Hispanic participants rather than the same size). Hispanic participants received weights of 1.

We estimated 9-month PFSD from the following Kaplan-Meier methods: (A) in the combined population with no weights (ie, “unweighted combined”), (B) in non-Hispanic White participants with no weights, (C) in non-Hispanic White participants with OW, (D) in Hispanic participants with no weights, and (E) in the combined population using weights (ie, “weighted combined”). As a sensitivity analysis, we explored change in weighted combined estimates when dividing OW by marginal odds of selection to create stabilized OW (analogous to stabilized inverse probability of treatment weights) [34]. Limits for 95% CIs of each quantity were obtained from 2.5th and 97.fifth percentiles of 2000 bootstrap iterations. We also calculated confidence limit differences (CLDs) to capture precision. Finally, we estimated 9-month PFSDs in Hispanic participants using outcome modeling and augmented odds weighting approaches [13,35] described in Appendix 3 to assess whether findings varied across analytic methods.

#### 2.4.3. Targeting mutant-type KRAS participants

We also used data from participants with wild-type KRAS to improve precision of effect estimates in participants with mutant-type KRAS. Because this target subgroup is defined by KRAS variant (a cEMM no matter what), inference-extending methods should yield biased estimates. Data included 513 participants with wild-type and 352 participants with mutant-type KRAS. For these analyses, we adjusted for biologic sex, age greater than 65 years, colon vs rectal cancer, presence of liver metastases, and performance status.

## 3. Results

### 3.1. Key demographic characteristics and overall results

Table 1 shows distributions of demographic and clinical variables in Hispanic and non-Hispanic White participants before and after OW. Wild-type vs mutant-type KRAS, the proportion of participants over age 65 years, and the proportion of female participants all differed between Hispanic and non-Hispanic White participants. Weighting eliminated differences and resulted in equal population sizes. Supplemental Table 1 provides information when targeting mutant-type KRAS participants. Table 2 shows results of analyses targeting each subgroup.

### 3.2. Hispanic participants

**Figure 3.**
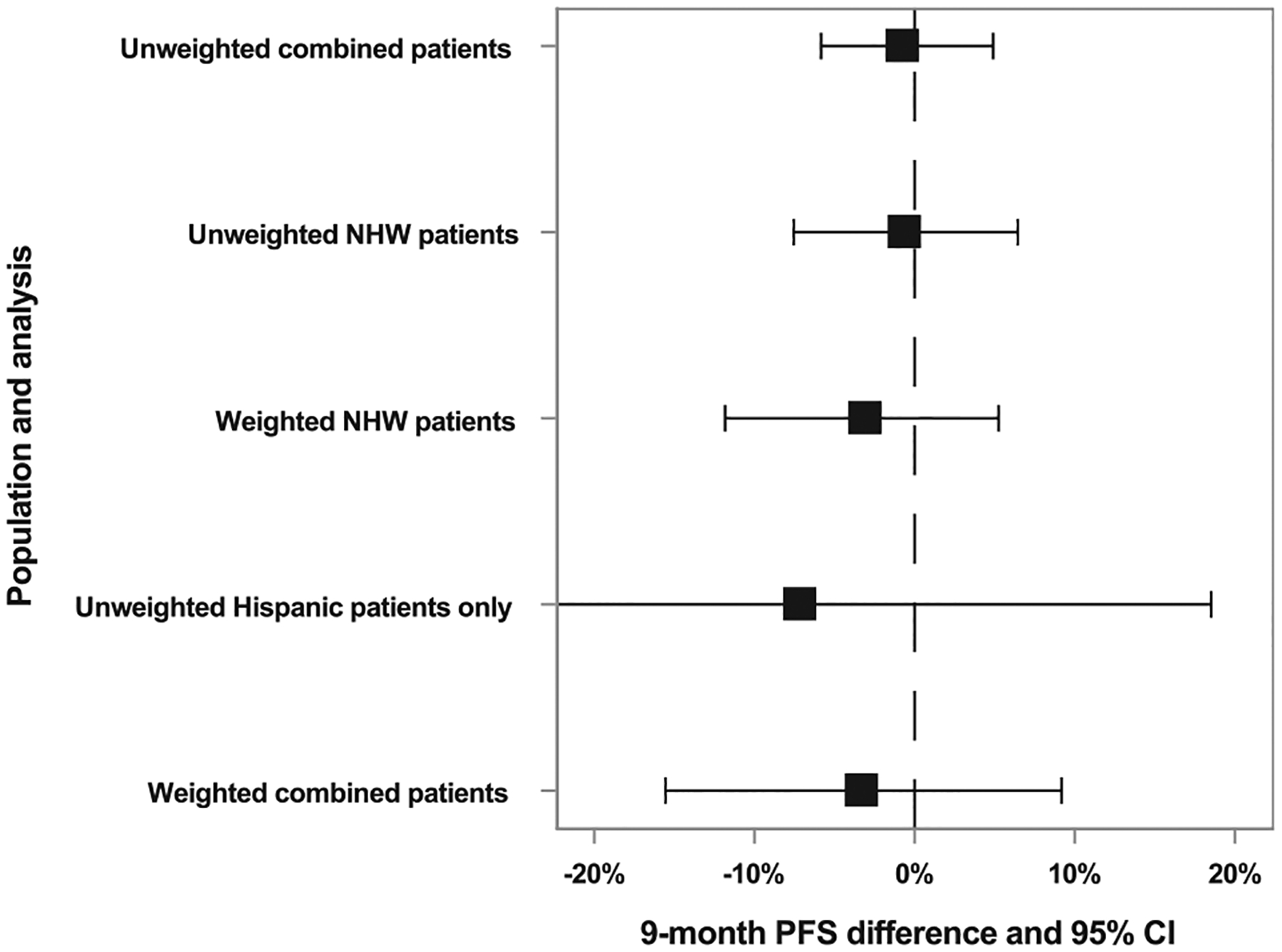
Estimates of the 9-month progression-free survival (PFS) difference analyzing unweighted combined patients, unweighted non-Hispanic white (NHW) patients, odds-weighted (OW) NHW patients, Hispanic patients, and the weighted combined patients. The 95% CIs are based on the 2.5th and 97.fifth percentiles of 2000 bootstrap iterations and the dashed line represents the null (9-month PFS difference of 0).

Fig 3 shows point estimates and 95% confidence limits for 9-month PFSD when targeting Hispanic participants. The Hispanic participant estimate was imprecise (9-month PFSD CLD: 51%) compared to the non-Hispanic White participant estimate (9-month PFSD CLD: 14%). After OW, the non-Hispanic White participant estimate was closer to the Hispanic participant estimate but less precise (9-month PFSD: −3.1%, 95% CI: −12%, 5.2%, CLD: 17%). Using outcome modeling (9-month PFSD: −3.3%, 95% CI: −13%, 6.5%, CLD: 20%) or doubly robust approaches (9-month PFSD: −1.8%, 95% CI: −10%, 6.3%, CLD: 16%) yielded similar results. Contrary to expectation, outcome modeling was less precise than weighting because outcome models required terms for time since randomization to capture time-varying outcome incidence.

Relative to the estimate in Hispanic participants only, the weighted combined estimate was closer to 0, had a CI half as wide (9-month PFSD: −3.7%, 95% CI: −16%, 9.2%, CLD: 25%), and the CI was entirely contained within the Hispanic subgroup estimate. It was less precise than the unweighted combined estimate (9-month PFSD CLD: 11%). With stabilized OW, the weighted combined estimate was similar with greater precision (9-month PFSD CLD: 17%).

### 3.3. Mutant-type KRAS participants

When targeting mutant-type KRAS participants, weighting changed the estimate in wild-type KRAS participants slightly but did not substantially alter precision. However, the increase in precision from the weighted combined vs the subgroup-only estimate was substantial (weighted combined PFSD CLD: 11%, mutant-type KRAS alone PFSD CLD: 16%). Weighted combined results were closer to 0 (9-month PFSD: −2.2%, 9% CI: −7.5%, 3.3%) than the mutant-type subgroup alone (9-month PFSD: −11%, 95% CI: −18%, −2.3%) or the full PRIME trial [21].

## 4. Discussion

We increased precision of estimates of effects of adding panitumumab to chemotherapy in Hispanic trial participants with metastatic colon or rectal cancer by weighting non-Hispanic White participants to resemble Hispanic participants. Alternatives like outcome modeling or augmented OW yielded similar results. When estimating subgroup effects, methods for extending inferences to external targets allow subgroup nonmembers to contribute to subgroup analyses without assuming homogeneity. Unfortunately, combined estimates are biased when key assumptions are violated (ie, imbalance in an unmeasured EMM between groups or group membership itself being a cEMM).

This is closely related to work on causal meta-analysis in multisite randomized trials [17,18], combining results in distributed networks [19], and synthesizing evidence for conditional average effects [36,37]. Two things make this approach attractive in subgroup analyses. First, directly combining data from subgroup members and nonmembers simplifies implementation. Second, differential missingness [38] and measurement error [39] are less likely between subgroups than between sites.

These methods possess major limitations. Like any model-based methods, they assume no measurement error, no selection bias from loss-to-follow-up or missing data, and correctly specified models [6,40]. In addition, as discussed previously, they work only when accounting for measured variables eliminates effect measure modification by the subgroup defining characteristic. When this is not the case (eg, wild-type KRAS participants) restricting analyses to subgroup members and accepting limited precision is the unbiased approach. Omitted variable bias in combined estimates will be larger in small subgroups when using IVW. Using IVW to target mutant-type patients, for example, generated a more precise weighted combined estimate farther from the mutant-type estimate (9-month PFSD: −1.4%, 95% CI: −6.9%, 4.0%). Minimally sufficient adjustment sets differ for each effect measure of interest [24] and methods to identify those sets have low power [41,42]. Finally, there is no established framework for reporting these results.

The utility of this approach in our example, where routinely measured tumor KRAS status drives heterogeneity and not all patients should be treated, is limited (KRAS-status specific estimates and KRAS-specific estimates in Hispanic patients are more relevant). If obtaining accurate information on the driver of heterogeneity is impossible or unreliable in real-world settings compared to the subgroup-defining characteristic; however, this approach can identify whether real-world subgroup membership could reasonably identify intervention candidates. In addition, if all patients will be treated (either because everyone benefits or because the intervention is population-wide), this approach can obtain more precise estimates of the impacts on disparities between groups and assess potential consequences of intervening in some groups before others.

Our analyses were simple for illustrative purposes. We did not use Bayesian approaches to combine estimates while incorporating uncertainty [43] around whether assumptions only hold for some nonmembers. We also focused on trials, though methods can be adapted to nonexperimental studies under no unmeasured confounding [20]. While we cannot share data, PRIME data is easily requested from Project Datasphere. Finally, while we did not preregister a protocol, future applications estimating clinically important effects should register protocols prespecifying subgroups and covariates (or algorithms for selecting covariates) [44].

## 5. Conclusion

Estimating effects in subgroups is a key part of epidemiologic research. Methods extending inferences to defined target populations can improve subgroup effect estimate precision under external consistency, exchangeability, and positivity. However, violations of those assumptions will yield biased estimates.

## Supplementary Material

1

Supplementary data

Supplementary data to this article can be found online at https://doi.org/10.1016/j.jclinepi.2025.111716.

## Figures and Tables

**Table 1.** Distributions of key potential effect measure modifying covariates in Hispanic participants and non-Hispanic white participants before and after applying odds weights

Potential effect measure modifier	Non-Hispanic white participants (*N* = 795)	Hispanic participants (*N* = 42)	Odds-weighted non-Hispanic white participants (*N* = 41.5)
Wild-type KRAS N (%)	479 (60%)	17 (40%)	17.3 (42%)
Over age 65 years N (%)	312 (39%)	12 (29%)	12.0 (29%)
Female N (%)	291 (37%)	22 (52%)	21.4 (51%)
Liver metastases N (%)	704 (89%)	39 (93%)	38.4 (92%)
Colon cancer N (%)	545 (69%)	21 (50%)	20.9 (50%)
ECOG 0 N (%)	444 (56%)	20 (47%)	19.9 (48%)
ECOG 1 N (%)	310 (39%)	21 (50%)	20.6 (50%)
ECOG 2 N (%)	41 (5%)	1 (2%)	1.0 (2.5%)

KRAS, Kristen rat sarcoma. ECOG, Eastern Cooperative Oncology Group.

**Table 2.** Nine-month progression-free survival difference (PFSD) estimates from the unweighted combined analysis, the nontarget subgroup members, the weighted nontarget subgroup members, the target subgroup members, and the weighted combined analysis with 95% CIs from the percentiles of 2000 bootstrap iterations for the target subgroups

Analysis	Targeting Hispanic participant subgroup		Targeting mutant-type KRAS subgroup	
	9-month PFSD (95% CI)	CLD	9-month PFSD (95% CI)	CLD
Unweighted combined analysis^a^	−0.7% (−5.8%, 4.9%)	11%	−0.2% (−5.3%, 5.1%)	10%
Nontarget participants only	−0.6% (−7.5%, 6.4%)	14%	6.8% (−1.7%, 15.6%)	17%
Weighted nontarget participants only	−3.1% (−12%, 5.2%)	17%	6.1% (−1.2%, 13.5%)	15%
Target subgroup participants only	−7.1% (−32%, 19%)	51%	−11% (−18%, −2.3%)	16%
Weighted combined analysis	−3.7% (−16%, 9.2%)	25%	−2.2% (−7.5%, 3.3%)	11%

PFSD, Progression-free survival difference (positive = panitumumab beneficial).

a Unweighted combined analyses differ due to including participants that were neither Hispanic nor non-Hispanic White in the analyses when targeting participants with wild-type KRAS tumor variants. Each analysis also used separate random seeds when bootstrapping, generating slight differences in confidence limits.

## Data Availability

The authors do not have permission to share data.

## What is new?

### Key findings

- Methods traditionally used to extend inferences from study samples to external target populations can be used to bolster precision of subgroup estimates without ignoring heterogeneity.
- These methods should be applied only when the characteristic defining the subgroup does not modify the treatment effect of interest conditional on measured covariates.

## What this adds to what is known?

- We describe the fundamental logic underlying this approach using a simple hypothetical example, use a simulation to explore different approaches for combining estimates, and showcase the potential for benefit and bias in real data from a randomized trial.

## What is the implication and what should change now?

- Researchers estimating treatment effects in small subgroups should consider applying these approaches in cases where subgroup membership is associated with, but not does itself directly modify, the treatment effect of interest.
- Resulting estimates should be interpreted with caution in case there are unknown or unmeasured sources of effect measure modification. Future research should examine formally incorporating into estimates the uncertainty surrounding the method’s assumptions.
