# Supplementary material for: Using methods to extend inferences to specific target populations to improve the precision of subgroup analyses
Source: J Clin Epidemiol. Author manuscript; Available in PMC 2026 May 21. (PMC13192252; doi:10.1016/j.jclinepi.2025.111716)
Supplement: 1 [file NIHMS2171261-supplement-1.docx]

Using Methods to Extend Inferences to Specific Target Populations to Improve the Precision of Subgroup Analysis

Supplemental Digital Content

**Page 2-Appendix 1:** Mathematical definitions for key terms

**Page 4-Appendix 2:** Simulation description and results

**Page 8-Appendix 3:** Outcome modeling and augmented odds weights approaches.

**Page 11-Supplemental Table 1:** Distributions of key potential effect measure modifying covariates in mutant-type KRAS participants and wild-type KRAS participants before and after applying odds weights.

**APPENDIX 1: MATHEMATICAL DEFINITIONS FOR KEY TERMS**

*Effect measure modification:* A binary variable *M* acts as an EMM for the risk difference of a binary exposure *X* on a binary outcome *Y* if it satisfies the inequality:

$\Pr\left( Y^{X=1} | M=1 \right)-\Pr\left( Y^{X=0} | M=1 \right)\neq\Pr\left( Y^{X=1} | M=0 \right)-\Pr\left( Y^{X=0} | M=0 \right)$,

where $\Pr\left( Y^{X=1} | M=1 \right)$ refers to the population’s probability of the outcome under treatment *X=1* conditional on *M=1*.

*Consistency:* Consistency is the assumption that, for each exposure level of interest x:

$\Pr\left( Y | X=x \right)=\Pr\left( Y^{X=x} \right)$,

Where $\Pr\left( Y | X=x \right)$ refers to the observed outcomes among patients that received treatment level x of the X variable, and $\Pr\left( Y^{X=x} \right)$ refers to the potential outcome if we assigned patients to level x of the X variable.

*Conditional effect measure modification:* A binary variable *M* acts as a conditional EMM for the risk difference of a binary exposure *X* on a binary outcome *Y* if it satisfies the following inequality under a set of covariates ***L*** :

$$\Pr\left( Y^{X=1} | M=1,\boldsymbol{L=l} \right)-\Pr\left( Y^{X=0} | M=1,\boldsymbol{L=l} \right)\neq$$

$$\Pr\left( Y^{X=1} | M=0,\boldsymbol{L=l} \right)-\Pr\left( Y^{X=0} | M=0,\boldsymbol{L=l} \right)$$

where ***L=l*** refers to a specific common distribution of covariates in ***L***.

*External consistency:* For a study population S=1 receiving a binary exposure X_study_, a target population S=0 receiving a binary exposure X_target_, and a binary outcome Y, external consistency means that for each individual *i* in the target population the probability of experiencing the outcome Y is the same for corresponding levels of X_study_ and X_target_. Mathematically, this means:

$\forall_{i}P\left( Y_{i}^{Xstudy=1} \right)=P\left( Y_{i}^{Xtarget=1} \right)$ and $\forall_{i}P\left( Y_{i}^{Xstudy=0} \right)=P\left( Y_{i}^{Xtarget=0} \right)$

*External exchangeability:* For a study population S=1, a target population S=0, a set of covariates ***L***, an exposure X, and a binary outcome Y, external exchangeability for the risk difference requires satisfying this equation:

$Pr\left( Y^{X=1} | S=1,\boldsymbol{L=l} \right)-Pr\left( Y^{X=0} | S=1,\boldsymbol{L=l} \right)= Pr\left( Y^{X=1} | S=0,\boldsymbol{L=l} \right)-Pr\left( Y^{X=0} | S=0,\boldsymbol{L=l} \right)$,

*External positivity:* For a study population S=1, a target population S=0, a set of covariates ***L***, an exposure X, and a binary outcome Y, external positivity for the risk difference requires satisfying this equation:

$IF\Pr\left( \boldsymbol{L=l}, S=0 \right)>0, THEN Pr\left( \boldsymbol{L=l} | S=1 \right)>0$,

Meaning that every necessary combination of covariates ***L*** present in S=0 must be present in S=1.

**APPENDIX 2: SIMULATION DESCRIPTION AND RESULTS**

We simulated 20,000 iterations of a 1,000 individual randomized controlled trial of a binary treatment X and a binary outcome Y. There were three other variables included in the simulation: SUB, C1, and C2. SUB was the subgroup-defining characteristic and was present in 5% of patients. Patients with SUB=1 had a 30% chance of having C1 as well as a 30% chance of having C2 (with those probabilities being independent). Patients with SUB=0 had a 60% chance of C1 and C2 instead. Treatment with X was completely random.

The probability of experiencing Y was defined as follows:

P(Y) = 0.1 + 0.1*C1 + 0.1*C2 + 0.2*C1*X + 0.1*C2*X

…meaning that both C1 and C2 were important effect measure modifiers of the effect of X on Y and making the expected average treatment effect in those with SUB=1 equal to 0.09 and the expected average treatment effect in those with SUB=0 equal to 0.18.

The full data generating mechanism is summarized below:

| Total N | 1,000 |
| --- | --- |
| P(X=1) | 0.50 |
| P(SUB=1) | 0.05 (base case)  0.95 (large subgroup scase) |
| P(C1=1 \| SUB=1) | 0.30 |
| P(C2=1 \| SUB=1) | 0.30 |
| P(C1=1 \| SUB=0) | 0.60 |
| P(C2=1 \| SUB=0) | 0.60 |
| P(Y=1) | P(Y) = 0.1 + 0.1*C1 + 0.1*C2 + 0.2*C1*X + 0.1*C2*X |

We compared two different approaches to estimating the treatment effect in patients with SUB=1:

1. Combined odds weights. In this approach, those with SUB=0 were weighted to resemble those with SUB=1 using odds weights, while those with SUB=1 were assigned odds weights of 1. The difference in the probability of Y was then calculated between treatment groups within this combined cohort.
2. Odds weighting followed by inverse variance weighting. In this approach, those with SUB=0 were weighted to resemble those with SUB=1 using odds weights and the SUB=1 and SUB=0 cohorts were analyzed separately resulting in two treatment effect estimates, one in the SUB=0 population after odds weighting and one in the SUB=1 population with no weighting. The two estimates were then combined using inverse variance weighting (i.e., taking a weighted average of the two estimates with weights equal to the inverse of each estimate’s variance and assigning that weighted average a variance equal to the inverse of the sum of the inverse variance weights).

We did not perform nested bootstraps within each individual simulation iteration to obtain standard errors and confidence limits; instead, we calculated standard errors and variances based on the standard deviations of the estimates across the 20,000 simulation iterations. This may nominally affect confidence interval coverage, means, and standard errors.

We calculated the proportion of the 95% confidence limits of the combined and inverse variance weighted estimates that contained the true value of 0.09 across all 20,000 simulation iterations. We also calculated overall means and standard errors based on the standard deviation of the combined and inverse variance weighted estimates.

We explored how these quantities changed when omitting C1, C2, or both variables from the odds weights. We then repeated the analyses in alternative simulation scenarios with 50% and 95% of patients having SUB=1.

**Results:**

| **Scenario and analysis** | **95% confidence interval coverage** | | **Mean estimate** | | **Standard error** | |
| --- | --- | --- | --- | --- | --- | --- |
|  | **Combined** | **IVW** | **Combined** | **IVW** | **Combined** | **IVW** |
| **Scenario: 5% SUB=1** |  |  |  |  |  |  |
| Weighting by C1 and C2 | 95.13% | 94.78% | 0.0897 | 0.0902 | 0.0603 | 0.0341 |
| Weighting by C2 only | 94.46% | 86.18% | 0.1047 | 0.1176 | 0.0601 | 0.0329 |
| Weighting by C1 only | 91.90% | 57.17% | 0.1199 | 0.1457 | 0.0593 | 0.0316 |
|  |  |  |  |  |  |  |
| **Scenario: 50% SUB=1** |  |  |  |  |  |  |
| Weighting by C1 and C2 | 94.90% | 94.75% | 0.0903 | 0.0903 | 0.0285 | 0.0280 |
| Weighting by C2 only | 91.75% | 92.88% | 0.1051 | 0.1023 | 0.0282 | 0.0277 |
| Weighting by C1 only | 81.61% | 86.45% | 0.1203 | 0.1140 | 0.0285 | 0.0279 |
|  |  |  |  |  |  |  |
| **Scenario: 95% SUB=1** |  |  |  |  |  |  |
| Weighting by C1 and C2 | 94.87% | 94.64% | 0.0915 | 0.0903 | 0.0730 | 0.0256 |
| Weighting by C2 only | 94.40% | 94.75% | 0.1052 | 0.0912 | 0.0696 | 0.0255 |
| Weighting by C1 only | 92.93% | 94.75% | 0.12072 | 0.0921 | 0.0728 | 0.0256 |

When all necessary covariates were included in the weighting model, combined and IVW approaches yielded similar estimates and similar 95% confidence interval coverage, though the combined approach was either slightly less (when the target subgroup was 50% of the population) or much less (when the target subgroup was 5% or 95% of the population) precise. When some necessary variables were omitted, the combined approach yielded less biased estimates with better confidence interval coverage at the cost of precision when the target subgroup was 5% of the population, but more biased estimates with worse confidence interval coverage when 50% or 95% of the study population were target subgroup members. The same trends were observed when we used outcome modeling to estimate the effect in those with SUB=1 using data from those with SUB=0.

**APPENDIX 3: OUTCOME MODELING AND DOUBLY ROBUST ESTIMATION**

Our primary analysis estimated the treatment effect in subgroup members by reweighting non-members based on potential effect-measure-modifying characteristics. The reweighting method assumes that we have correctly specified the prediction model we used to create the weights. Outcome prediction and doubly robust methods are alternative approaches to estimating treatment effects in external target populations that can also be applied to estimate treatment effects in target subgroup populations using treatment, covariate, and outcome data from non-target subgroup individuals and may be more precise (at least in cases with continuous outcomes or binary outcomes with no loss to follow-up). We explored both methods to understand the extent to which they might alter the treatment effect estimate in Hispanic patients when drawing on data from non-Hispanic White patients.

**Method 1: Outcome modeling**

Our main analysis estimated the effect of adding panitumumab to FOLFOX regimens on 9-month progression-free survival. Because some patients were lost to follow-up prior to this time point (52/795 non-Hispanic White patients), with many of them being lost early on during follow-up, fitting a cross-sectional model to predict progression-free survival among the complete cases did not seem appropriate. Instead, we created longitudinal data with 10-day observation periods covering day 0-1 to day 269-270 and fit two multivariable logistic regression models among the non-Hispanic White patients (one for each treatment group) predicting the probability of experiencing progression or mortality within each 10-day increment based on tumor KRAS type, sex, baseline performance status, age over 65, time since baseline (with linear and quadratic terms), colon vs rectal cancer, and the presence of liver metastases. We then used the estimated coefficients from these models to predict the probability of the progression-free survival until day 270 among the Hispanic trial participants when treated with FOLFOX alone or with FOLFOX and panitumumab then subtracted them to obtain the difference in progression-free survival. We obtained the 2.5^th^ and 97.5^th^ confidence limits of this difference from 2,000 bootstrap iterations.

**Method 2: Augmented odds weighting**

Despite the similarity between our odds-weighted (9-month progression free survival difference in Hispanic participants based on data from non-Hispanic white participants: -3.1%, 95% CI: -11.8%, 5.2%) and outcome-modeled (9-month progression-free survival difference in Hispanic participants based on data from non-Hispanic white participants: -3.4%, 95% CI: -13.4%, 6.5%) estimates, we were also interested in exploring a doubly robust approach that would yield unbiased results if either one of the weighted or outcome models were correctly specified. The simplest approach is an adaptation of the augmented inverse probability weights that can be used as a doubly robust estimator for internal validity. While this process is straightforward in the absence of loss to follow-up, the fact that some patients were censored complicates it somewhat by requiring the use of inverse probability of censoring weights when calculating residual errors based on weights. Our approach was as follows:

1. Fit the outcome model described above among the non-subgroup members.
2. Use that outcome model to predict the probability of the outcome for each target population member under each treatment option of interest.
3. Within each treatment condition, sum the predicted probabilities of the outcome.
4. Create external validity weights (in our case, odds weights).
5. Use the outcome model to predict the probability of the outcome among the non-subgroup members.
6. Calculate the **residual error** in the predictions among the non-subgroup members, making sure to use the prediction model related to their factual outcome (e.g., if the model predicted a 10% chance of survival, and they survived, the residual error = 0.90; if they had died, the residual error = -0.10).
7. **Sum** these residual errors with each error receiving a weight equal to the associated individual’s odds weights multiplied by the inverse probability of remaining uncensored through day 270.
8. Within each treatment condition, **sum** the predicted probabilities and the residual errors.
9. **Divide** by the total number of individuals in the target subgroup. This results in two proportions representing the predicted probabilities of progression-free survival within the target subgroup, one for each treatment group.
10. **Subtract** these progression free survival predictions from one another to obtain your estimated treatment effect.

We calculated confidence limits is via the 2.5^th^ and 97.5^th^ percentiles of estimates across 2,000 bootstrap iterations.

**Supplemental Table 1:** Distributions of key potential effect measure modifying covariates in wild-type KRAS participants and mutant-type KRAS participants before and after applying odds weights.

| Potential effect measure modifier | Wild-type KRAS participants (N=513) | Mutant-type KRAS participants (N=352) | Odds-weighted wild-type KRAS participants (N=352.6) |
| --- | --- | --- | --- |
| Wild-type KRAS N (%) | 513 (100%) | 0 (0%) | 352.6 (100%) |
| Over age 65 N (%) | 195 (38%) | 134 (38%) | 136.1 (39%) |
| Female N (%) | 184 (36%) | 143 (41%) | 144.5 (41%) |
| Liver metastases N (%) | 453 (88%) | 316 (90%) | 316.1 (90%) |
| Colon cancer N (%) | 326 (64%) | 255 (72%) | 256.9 (73%) |
| ECOG 0 N (%) | 297 (58%) | 184 (52%) | 184.3 (52%) |
| ECOG 1 N (%) | 188 (37%) | 153 (43%) | 154.3 (44%) |
| ECOG 2 N (%) | 28 (5%) | 15 (4%) | 14.1 (4%) |
